# Transcriptional profiling of long noncoding RNAs associated with leaf-color mutation in *Ginkgo biloba* L

**DOI:** 10.1186/s12870-019-2141-z

**Published:** 2019-11-29

**Authors:** Yaqiong Wu, Jing Guo, Tongli Wang, Fuliang Cao, Guibin Wang

**Affiliations:** 1 Co-Innovation Center for Sustainable Forestry in Southern China, Nanjing Forestry University, 159 Longpan Road, Nanjing, 210037 China; 2 Department of Forest and Conservation Sciences, Faculty of Forestry, The University of British Columbia, Vancouver, V6T 1Z4 Canada

**Keywords:** Leaf-color mutation, Differentially expressed lncRNAs, Target genes, Functional analysis

## Abstract

**Background:**

Long noncoding RNAs (lncRNAs) play an important role in diverse biological processes and have been widely studied in recent years. However, the roles of lncRNAs in leaf pigment formation in ginkgo (*Ginkgo biloba* L.) remain poorly understood.

**Results:**

In this study, lncRNA libraries for mutant yellow-leaf and normal green-leaf ginkgo trees were constructed via high-throughput sequencing. A total of 2044 lncRNAs were obtained with an average length of 702 nt and typically harbored 2 exons. We identified 238 differentially expressed lncRNAs (DELs), 32 DELs and 49 differentially expressed mRNAs (DEGs) that constituted coexpression networks. We also found that 48 *cis*-acting DELs regulated 72 target genes, and 31 *trans*-acting DELs regulated 31 different target genes, which provides a new perspective for the regulation of the leaf-color mutation. Due to the crucial regulatory roles of lncRNAs in a wide range of biological processes, we conducted in-depth studies on the DELs and their targets and found that the chloroplast thylakoid membrane subcategory and the photosynthesis pathways (ko00195) were most enriched, suggesting their potential roles in leaf coloration mechanisms. In addition, our correlation analysis indicates that eight DELs and 68 transcription factors (TFs) might be involved in interaction networks.

**Conclusions:**

This study has enriched the knowledge concerning lncRNAs and provides new insights into the function of lncRNAs in leaf-color mutations, which will benefit future selective breeding of ginkgo.

## Background

Only 1 to 2% of the total RNAs produced by eukaryotic cells during transcription are encoded to produce proteins, and the remaining RNAs are called noncoding RNAs (ncRNAs). ncRNAs play important roles in cells, such as rRNAs and tRNAs in protein synthesis, snRNAs in the splicing of nascent RNA, and microRNAs, siRNAs and piRNAs in inhibiting gene expression [1]. Among ncRNAs, there is a widely distributed class of ncRNA transcripts with lengths greater than 200 nucleotides and no protein-encoding function, named long noncoding RNAs (lncRNAs) [2–5]. Most lncRNAs are transcribed by RNA polymerase II and have a structure similar to that of mRNA, such as 5′ caps and 3′ poly (A) tails [6, 7]. According to the genomic location of lncRNAs relative to neighboring genes, lncRNAs can be divided into five classes: sense lncRNA, antisense lncRNA, intergenic lncRNA (lincRNA), intronic lncRNA, and bidirectional lncRNA [8]. Moreover, lncRNAs can be classified into signals, decoys, guides and scaffolds based on molecular function [3, 9].

LncRNAs can be dynamically expressed during differentiation, and different mature lncRNAs can be formed by polyadenylation and different alternative splicing events, allowing the same gene to form different lncRNA transcripts [10]. LncRNA is universally transcribed in eukaryotic cells and distributed in the cytoplasm, organelles and nucleus but mainly in the nucleus. LncRNA was first identified in a sequencing analysis of mice in 2002 [11]. Currently, the functional mechanisms of lncRNA in humans and animals have been studied in great depth, especially in terms of diseases. In recent years, with the continuous improvement of bioinformatics technology, including high-throughput sequencing technology and other biological technologies, research on plant lncRNAs has developed rapidly and received increasing attention. Currently, lncRNAs have been widely identified in plants such as *Arabidopsis thaliana* [12], *Zea mays* [13], *Salvia miltiorrhiza* [14], and *Populus* [15]. LncRNAs can affect a series of biological processes, such as epigenetic regulation, cell cycle regulation, cell differentiation regulation and secondary metabolite synthesis, by regulating the level of target genes [16–18].

Ginkgo (*Ginkgo biloba* L.) is a well-known relict plant that originates from China and has been described as a “living fossil” [19]. As a multifunctional tree species, ginkgo has important economic and medicinal values [20] and has attracted researchers’ attention with many studies have been reported on the origin and evolution, cytology, molecular biology, tree breeding and medicinal value of ginkgo [19, 21–25]. Ginkgo is also a popular ornamental species and widely cultivated worldwide [24]. However, there are few studies on its ornamental characteristics [26]. Leaf color is an important trait of ginkgo as a landscape plant. The most attractive ornamental feature of ginkgo is its golden leaves in autumn [26]. The yellow color mutant identified in ginkgo showed the phenotypic trait of yellow leaves for the entire leaf development period and had a longer foliage period than common ginkgo [27]. Thus, the mutant not only possesses an excellent ornamental value but also provides an ideal material to study the genetic control of the leaf pigment synthesis.

Previous studies have provided an understanding of the protein-coding genes involved in leaf-color mutation [26, 27], but the role of lncRNAs in the yellow-leaf mutation has rarely been reported. In this study, normal green leaves and mutant yellow leaves of ginkgo were used as research materials to investigate their regulatory mechanism of lncRNAs in leaf-color mutation. Our objectives are to (1) establish lncRNA libraries, identify and characterize the putative lncRNAs expressed; (2) construct a coexpression network for differentially expressed lncRNAs (DELs) and differentially expressed mRNAs (DEGs); (3) predict the target genes of *cis*- and *trans*-acting lncRNAs and their functions; and (4) perform correlation analysis between lncRNAs and transcription factors (TFs) in ginkgo leaves. These findings will provide a scientific foundation for further research on the potential function of leaf-color mutations and benefit the future selective breeding and cultivation of ginkgo.

## Results

### RNA sequencing and identification of lncRNAs in ginkgo leaves

To obtain comprehensive lncRNA transcripts (Additional file 3: data S1), we used six (three yellow-colored leaves (YL) and three normal green-colored leaves (GL)) cDNA libraries by RNA sequencing (RNA-seq) on the Illumina HiSeq X platform. After paired-end sequencing, there was a total of 493.71 M raw reads generated from GL and 484.42 M from YL (Table 1). The raw sequencing data were submitted to the Short Reads Archive (SRA) database under the accession number SRP182122. After the low-quality sequences were filtered out, approximately 931.52 M clean reads were generated from the six libraries with an average GC content of 42.89%. Among them, more than 93% of the sequences were located on the reference ginkgo genome.

**Table 1:** Quality of the sequencing data

Sample	Raw reads	Raw bases	Clean reads	Clean bases	Valid bases	Q30	GC
Gb_GL1	165.33 M	24.80 G	155.75 M	22.39 G	90.30%	92.07%	43.17%
Gb_GL2	164.41 M	24.66 G	154.79 M	22.27 G	90.32%	92.47%	43.22%
Gb_GL3	163.97 M	24.60 G	154.84 M	22.41 G	91.11%	92.79%	42.97%
Gb_YL1	165.47 M	24.82 G	156.14 M	22.38 G	90.16%	92.13%	42.69%
Gb_YL2	165.07 M	24.76 G	156.08 M	22.58 G	91.19%	92.43%	42.55%
Gb_YL3	163.88 M	24.58 G	153.92 M	22.03 G	89.61%	91.69%	42.74%

**Fig. 1:**
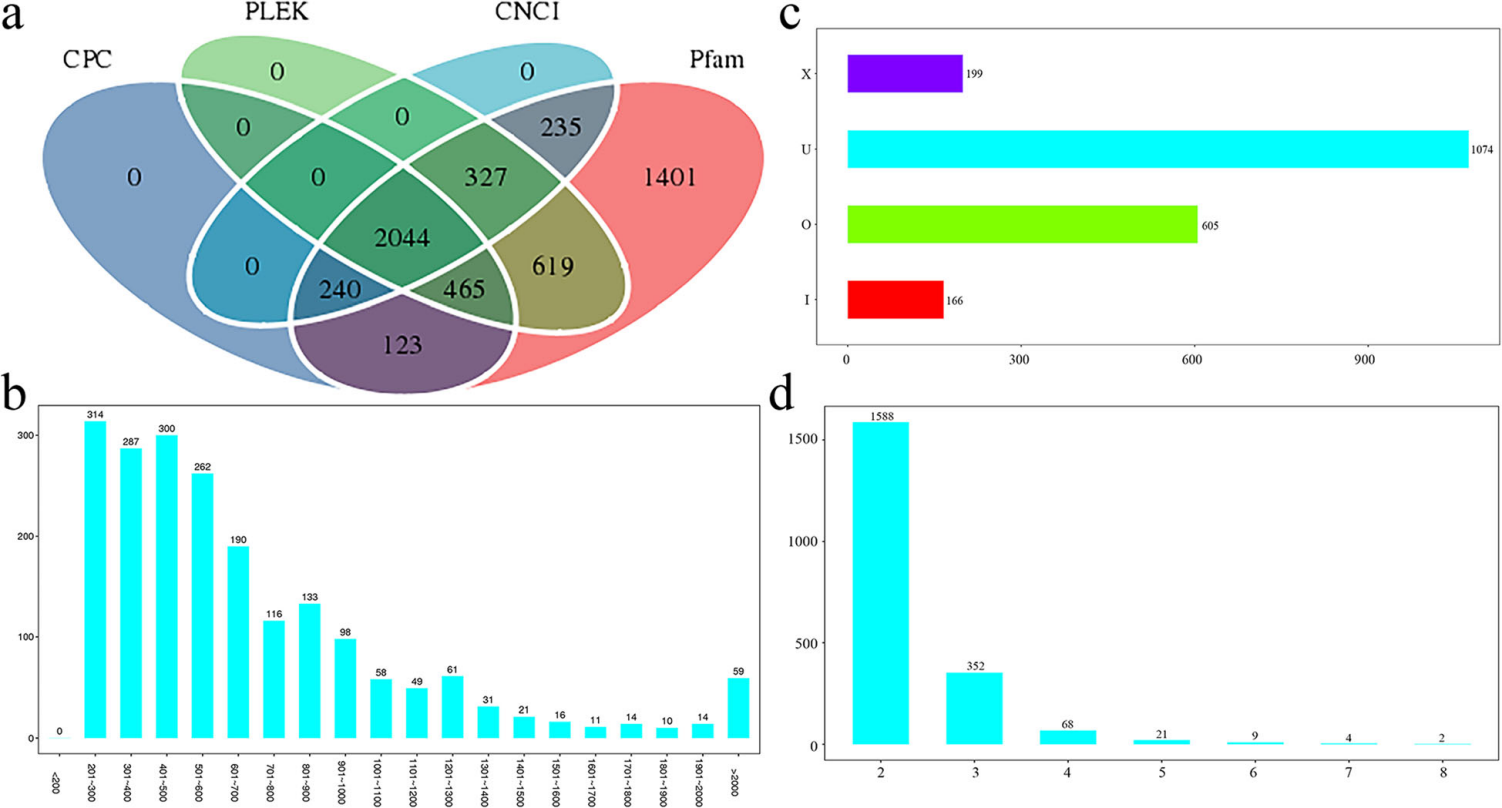
Summary of the characteristic long noncoding RNAs (lncRNAs) in ginkgo. **a** Venn diagram showing the numbers of candidate lncRNAs in ginkgo according to the Coding Potential Calculator (CPC) and Coding-Non-Coding Index (CNCI) as well the Protein Families (Pfam) and predictor of long noncoding RNAs and messenger RNAs based on an improved k-mer scheme (PLEK) databases. **b** LncRNA sequence length distribution. The lncRNA number is on the vertical axis, and the lncRNA length range is on the horizontal axis. **c** Statistical chart of lncRNA types. Types of lncRNAs on the vertical axis and number of lncRNAs on the horizontal axis. U: intergenic lncRNA; I: intronic lncRNA; X: antisense lncRNA; and O: sense-overlapping lncRNA. **d** Statistical graph of exon number of lncRNAs. The number of lncRNAs is on the vertical axis, and the number of exon(s) contained in lncRNAs is on the horizontal axis

LncRNA is a class of RNA molecules that have lengths over 200 bp and no protein-coding ability. Therefore, the coding potential of transcripts is predicted to determine whether the transcripts are lncRNAs. The candidate lncRNAs were further screened by using Coding Potential Calculator (CPC) analysis, Coding-Non-Coding Index (CNCI) analysis, Protein Families (Pfam) protein domain analysis and predictor of long noncoding RNAs and messenger RNAs based on an improved k-mer scheme (PLEK) analysis methods (Fig. 1a). After the candidate lncRNAs were screened, 2044 lncRNA sequences were finally predicted (≥ 200 bp). The average length of the lncRNA sequences was 702, the N50 was 840, and the total length of all lncRNA sequences was 1,436,133. The newly predicted lncRNA length distribution indicates that the number of lncRNAs between 201 and 300 bp was at most 314, and the number of lncRNAs between 1801 and 1900 bp was only 10 (Fig. 1b). Moreover, lncRNAs can be classified into four types according to their location in relationship with known protein-coding transcripts: intergenic lncRNA (lincRNA, U), intronic lncRNA (I), antisense lncRNA (X), and sense-overlapping lncRNA (O); the results show that the number of U-type lncRNAs was the highest (1074), nearly 6.5 times that of I-type lncRNAs (Fig. 1c). The number of exons predicted in lncRNAs is shown in Fig. 1d, indicating that lncRNAs primarily harbored two exons. In addition, since some lncRNAs can act as precursors to known miRNAs in plants, we aligned miRNA precursors with the lncRNA sequences we identified. Six novel lncRNAs were identified as precursors to known miRNAs. The results showed that TCONS_00021802 had the most miRNA precursors, while TCONS_00016273 had the fewest miRNA precursors.

### LncRNAs expression level analysis

**Fig. 2:**
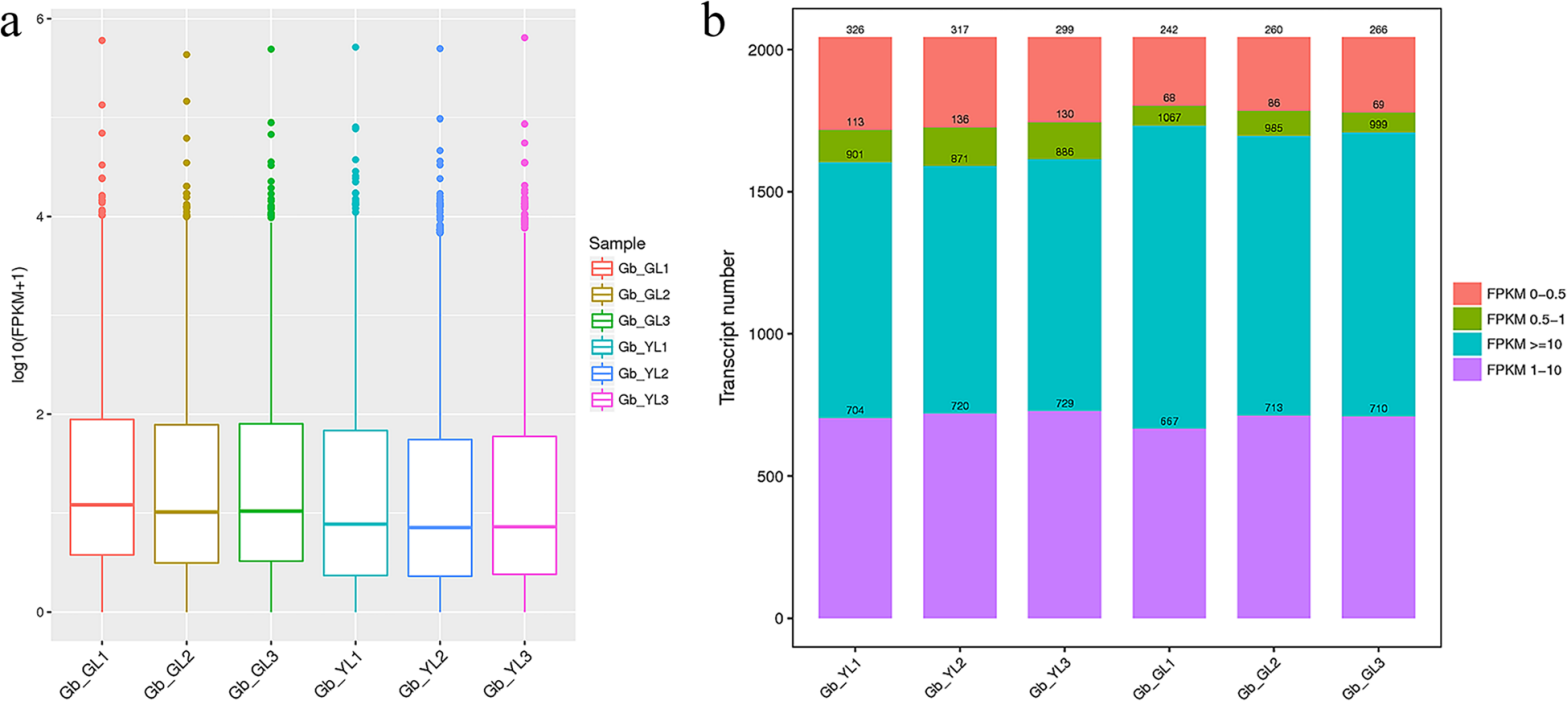
Long noncoding RNAs (lncRNAs) expression level analysis between GL and YL group in ginkgo. **a** Boxplot of fragments per kilobase per million reads (FPKM) values; **b** Transcript expression within each sample. The different colors of the FPKM expression distribution map represent different ranges of FPKM values

The expression levels of 2044 lncRNA transcripts were estimated via their fragments per kilobase per million reads (FPKM, Additional file 4: data S2). The results showed that these lncRNA expression levels were different, as shown in a boxplot (Fig. 2a). The average lncRNA expression levels of the YL group are significantly lower than those of the GL group (F = 34.180, *p* = 0.004). The results of interval expression values showed that the transcript number and distribution of gene expression values were different between YL and GL groups (Fig. 2b). Most transcripts had FPKM values greater than or equal to 10, whereas fewest number of transcripts had FPKM values between 0.5 and 1. The significance analysis results indicated that the YL group had more numbers of transcripts in FPKM 0–0.5 (F = 29.252, *p* = 0.006) and FPKM 0.5–1 (F = 33.155, *p* = 0.005), and less numbers in FPKM ≥10 (F = 23.957, *p* = 0.008) than the counterpart.

### Identification and validation of DELs between GL and YL

**Fig. 3:**
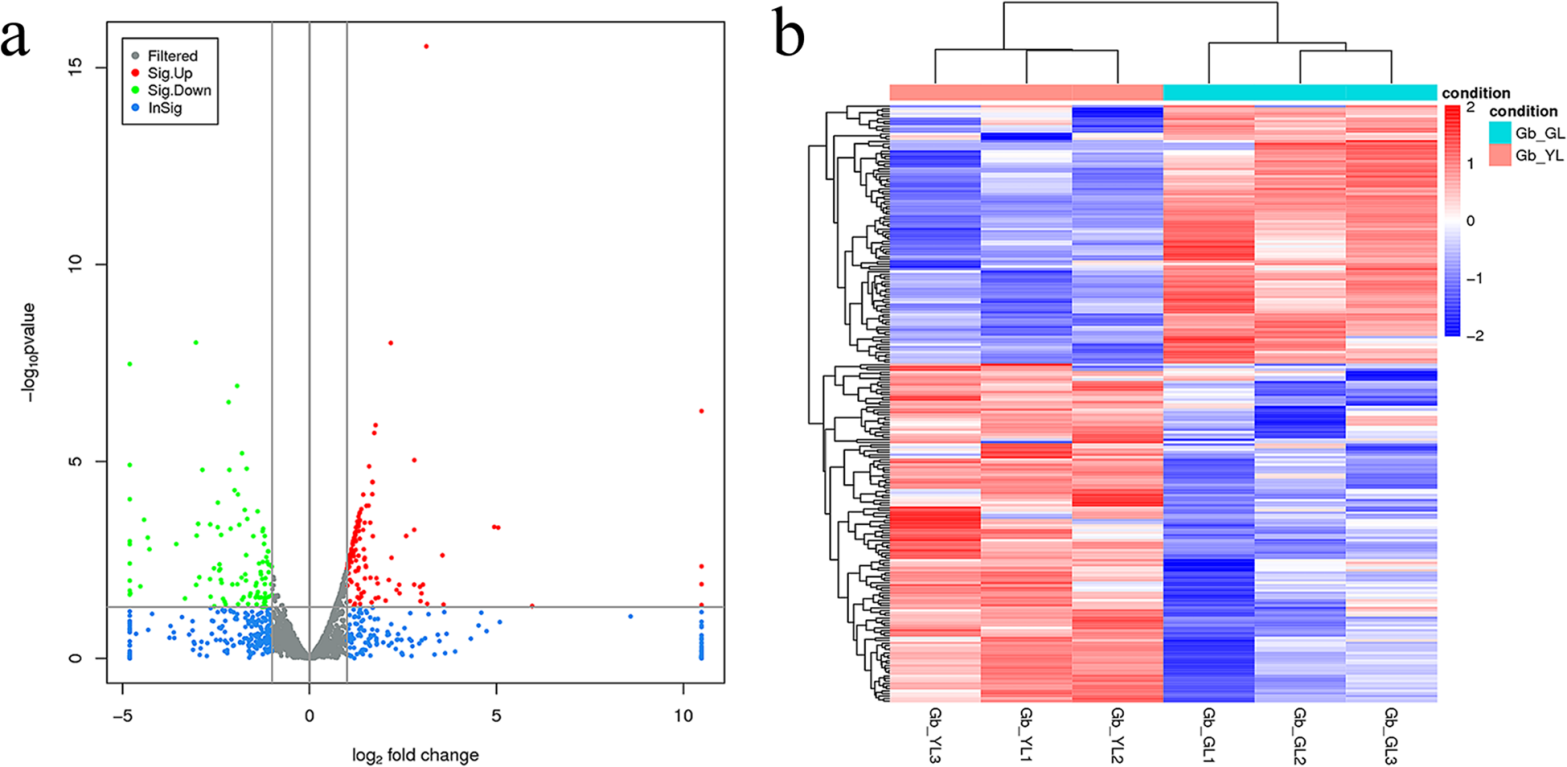
Differentially expressed lncRNAs (DELs) in ginkgo (Gb_GL and Gb_YL). **a** represents the differential volcano plot, the x-axis represents log2 fold change, and the y-axis represents log10 *p*-value. *P*-value < 0.05 and |log2 fold change| > 1; **b** Heat map clustering results of different groups. Red indicates high long noncoding RNA (lncRNA) expression, and blue indicates low lncRNA expression

A total of 238 DELs were screened according to the results of significance test (Additional file 5: data S3) between the GL and the YL groups. Among these DELs, 135 were significantly upregulated and 103 were significantly downregulated in the YL compared with GL group (Fig. 3). Moreover, there were more DEGs (1361, Additional file 6: data S4) than DELs. The overall distribution of DELs is depicted in a volcano plot. The red color represents the significantly upregulated lncRNAs, and the green color indicates significantly downregulated lncRNAs in the YL group (Fig. 3a). It can be seen from the heat map clustering that the same kind of samples appear in the same cluster by clustering analysis, and the genes in the same cluster may have similar biological functions (Fig. 3b).

To further verify the transcriptional patterns of DELs from the RNA-seq analysis, ten DELs were randomly selected and examined using quantitative real-time PCR (qRT-PCR) at the expression level. Although the expression multiples of several DELs verified by qRT-PCR were not completely the same as those of the FPKM values, the expression levels of these 10 DELs screened from the qRT-PCR analysis were consistent with those deduced from the FPKM values (Additional file 2: Figure S1, Additional file 5: data S3). Hence, these results indicated that the transcriptomic analysis results were reproducible and reliable, and would be useful for further studies of the lncRNAs functions (especially DELs) in GL and YL of ginkgo.

### DEL and DEG coexpression analysis

**Fig. 4:**
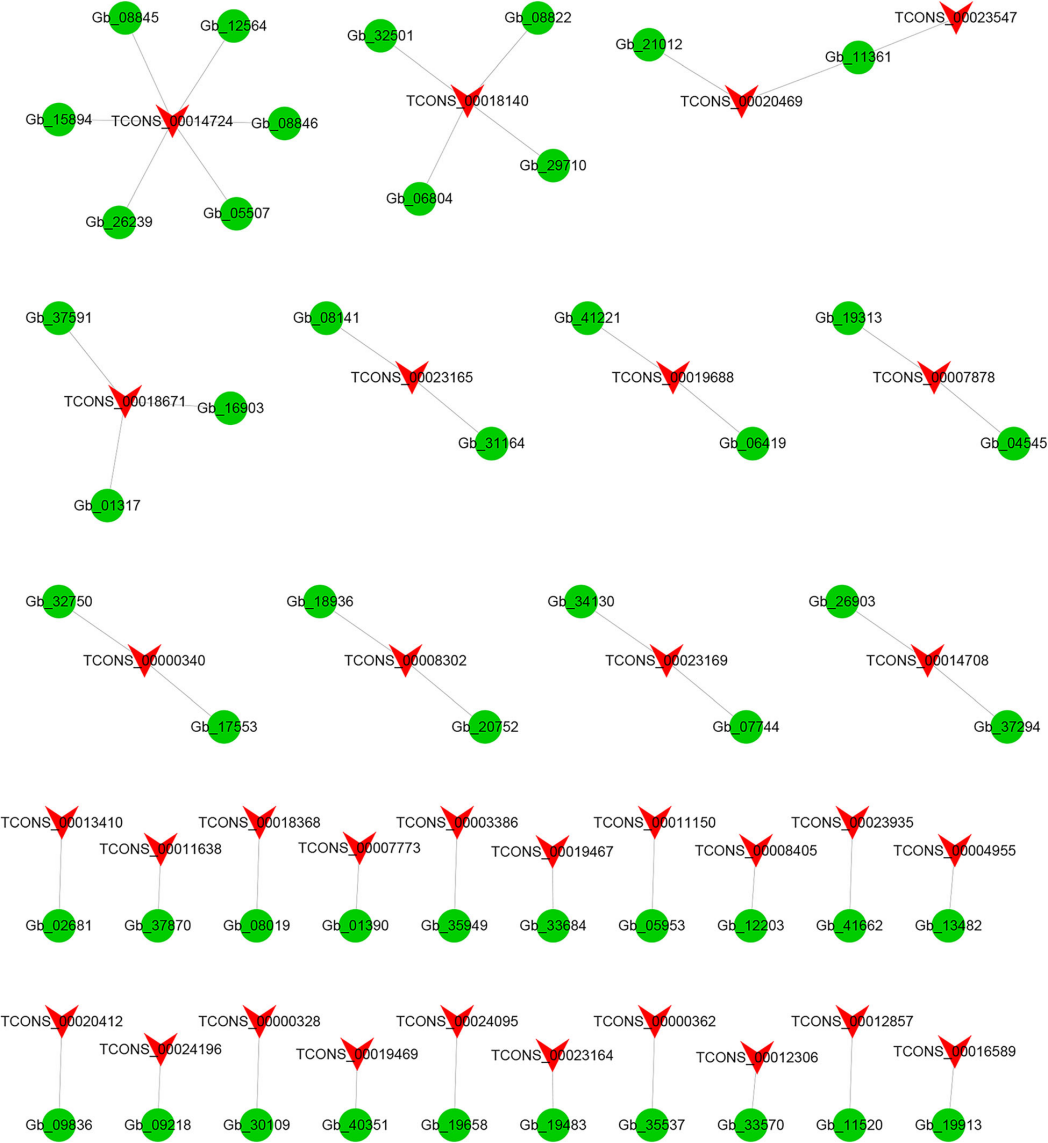
The coexpression network diagram of differentially expressed lncRNAs (DELs) and differentially expressed mRNAs (DEGs). The red arrow node represents DELs, and the green circular node represents DEGs

To further explore the functional relationship between DELs and DEGs, we constructed a coexpression network for DELs and DEGs in Fig. 4. The results showed that a total of 32 DELs and 49 DEGs constituted coexpression networks. The networks provide candidate DELs related to pigment function. Among them, TCONS_00014724 and six DEGs have a coexpression network. TCONS_00018140 and four DEGs (Gb_32501, Gb_08822, Gb_29710 and Gb_06804) exhibited coexpression patterns. The Gb_11361 gene was involved in the ko00195 photosynthesis pathway and was designated as photosystem I P700 chlorophyll a apoprotein A1 according to SwissProt. Gb_11361 was coexpressed with two DELs (TCONS_00020469 and TCONS_00023547). Most DELs and DEGs might have one-to-one coexpression patterns.

### Functional analysis of DEL target genes

**Fig. 5:**
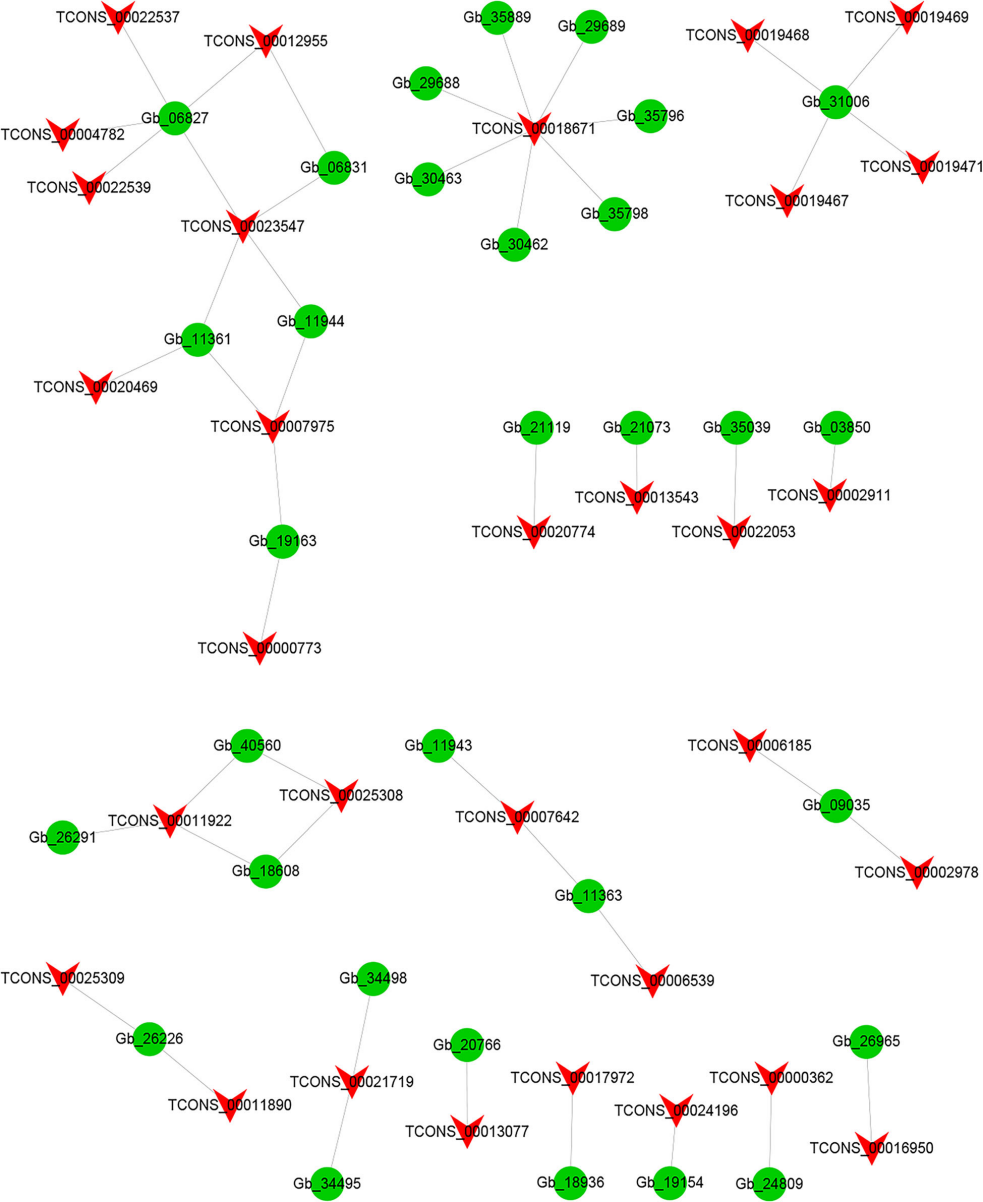
Analytical network diagram (Top50) of *trans*-acting differentially expressed lncRNAs (DELs) and differentially expressed mRNAs (DEGs). The red arrow node represents DELs, and the green circular node represents DEGs

To investigate the potential function of DELs, we predicted the candidate targets of *cis*- and *trans*-acting lncRNA. For the *cis*-acting lncRNAs, we searched for all coding genes in the upstream and downstream 100 kb range of DELs. These genes and lncRNAs with significant coexpression intersected. The results showed that 48 DELs may regulate 72 target genes within a range of 100 kb. These genes that are genomically neighboring and coexpressed in expression patterns are likely to be regulated by lncRNAs. For *trans*-acting lncRNAs (Fig. 5), the results of the lncRNA and mRNA analyses indicated that there were 31 different DELs that regulated 31 different target genes. Interestingly, four different lncRNAs (TCONS_00019467, TCONS_00019468, TCONS_00019469, and TCONS_00019471) have the same target gene Gb_31006. Another *trans*-acting lncRNA called TCONS_00018671 has seven target genes (Fig. 5). Nine DELs demonstrated one-to-one associations with nine DEGs.

**Fig. 6:**
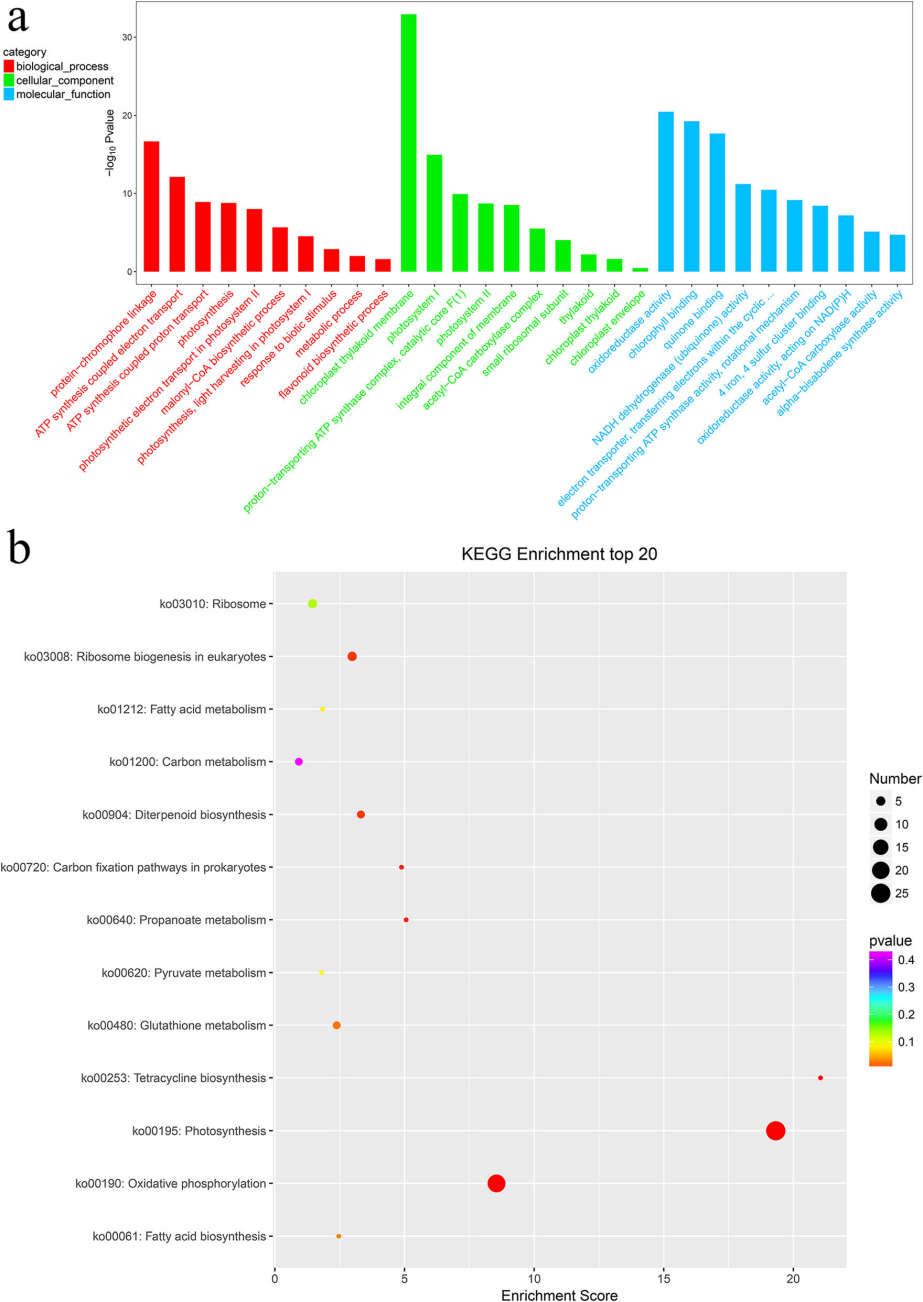
Enrichment analysis of lncRNA targets. **a** Gene Ontology (GO) enrichment analysis of long noncoding RNA (lncRNA) targets. **b** Kyoto Encyclopedia of Genes and Genomes (KEGG) pathway analysis of lncRNA targets

To further explore the potential biological function of lncRNAs, we performed Gene Ontology (GO) annotation analysis on *cis*- and *trans*-targets of lncRNAs (Fig. 6a). GO analysis results revealed that lncRNA target genes were categorized according to biological process (BP), cellular component (CC), and molecular function (MF). The protein-chromophore linkage and ATP synthesis coupled electron transport subcategories were enriched in the BP category. In the CC category, the chloroplast thylakoid membrane subcategory was the most enriched, followed by photosystem I. The oxidoreductase activity, chlorophyll binding and quinone binding were enriched in the MF category. We also analyzed the predicted target genes of lncRNAs using the Kyoto Encyclopedia of Genes and Genomes (KEGG) pathway database (Fig. 6b). The most highly represented pathways included photosynthesis (ko00195) and oxidative phosphorylation (ko00190).

### Correlation analysis of lncRNAs and TFs

**Fig. 7:**
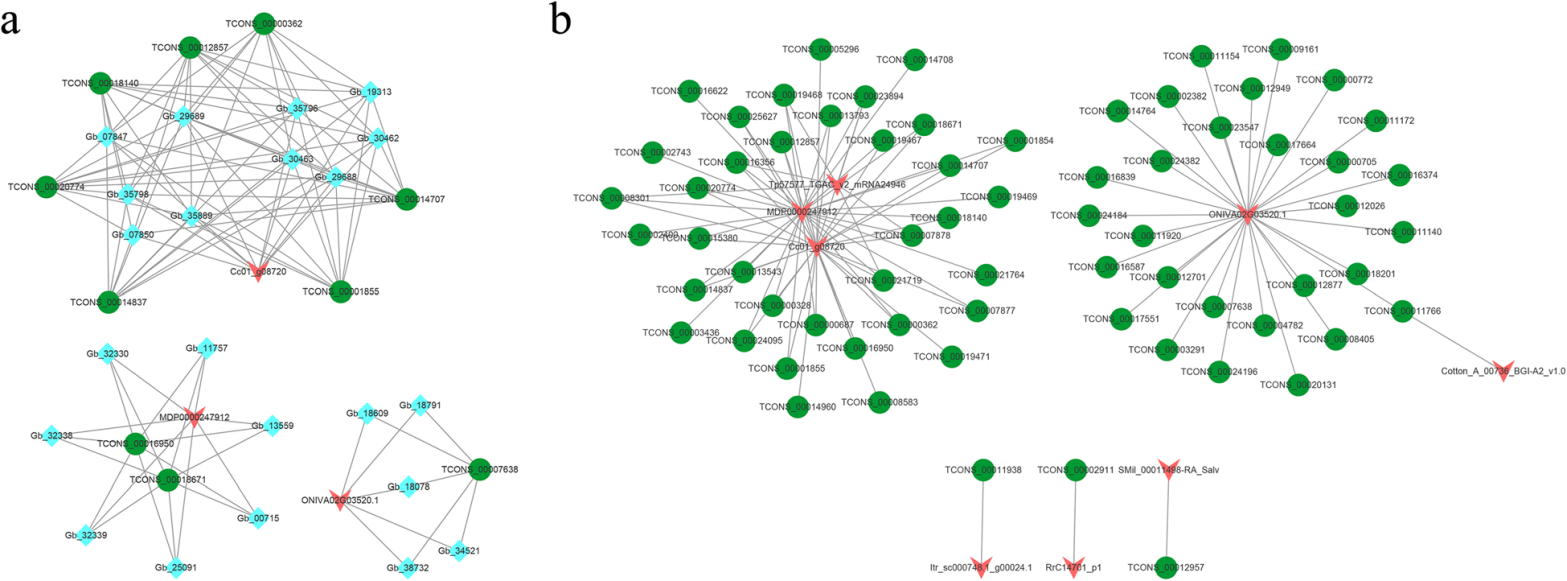
Interaction network diagrams. **a** Interaction networks of lncRNAs, mRNAs and transcription factors (TFs). **b** Interaction networks of lncRNAs and TFs. Red nodes represent TFs, green nodes represent lncRNAs, and blue nodes represent mRNAs

Because TFs play important roles in the network of leaf-color mutation genes, we identified TFs including MYB-related, bHLH, HSF, and others through a correlation analysis of lncRNAs and TFs. To further reveal the potential functions of DELs, we observed multiple pairs of lncRNA-TF relationships for each lncRNA. Each lncRNA-TF relationship is the result of the enrichment of multiple mRNAs. The results showed that the whole network constituted by these connections was divided into three clusters, including one large network and two relatively small networks (Fig. 7a). In the present study, we determined that TCONS_00007638 (lncRNA) interacts with ONIVA02G03520.1 (MYB-related TF) and five other mRNAs. Seven DELs, ten mRNAs and one TF have a relatively complex interaction network (Fig. 7a). Moreover, we also focused on the TFs targeted by lncRNAs in ginkgo. A total of 8 DELs and 68 TFs have interaction networks (Fig. 7b). ONIVA02G03520.1 (MYB-related TF) and 29 different lncRNAs have an interaction network. Itr_sc000748.1_g00024.1 (MYB-related TF) and TCONS_00011938, RrC14701_p1 (HSF TF) and TCONS_00002911, and SMil_00011498-RA_Salv (bHLH TF) and TCONS_00012957 have interaction networks.

## Discussion

LncRNAs play an important role in diverse biological processes and have been widely studied in recent years [12, 15, 28, 29]. Nevertheless, lncRNAs remain poorly understood in the context of leaf-color mutation and pigment formation in ginkgo. Leaf-color mutation is different pigment formation result characterized by a series of transitions that are coordinated by a network of interacting genes and pathways. Herein, we have constructed coexpression networks for DELs and DEGs, predicted the target genes of lncRNAs, and performed correlation functional research in leaf-color mutation leaves. Our study not only enriched the knowledge of lncRNAs but also provided new insights into the potential functions of lncRNAs in plants. These RNA-seq data might provide molecular targets that assist in the selective breeding and production of yellow-leaf ginkgo.

Bioinformatics technology for transcriptome analyses has been rapidly improved, and many lncRNAs have been identified in plants. For example, 9686 lncRNAs were found in Norway spruce [30]. Several studies have shown that lncRNAs are similar to mRNA-encoding proteins, but the number of lncRNA transcripts is lower than that of mRNA transcripts, and lncRNAs usually exist in the nucleus [31, 32]. Our study has confirmed Cui et al. [33] finding that the lncRNA expression levels in ginkgo leaves are lower and shorter in length than those of the coding genes. Similar findings were reported in previous studies [31, 32], and this conclusion was universal in plants [33]. These common factors may indicate the essential regulation of lncRNAs during growth, development and evolution [28]. In addition, ginkgo lncRNAs contained fewer exons (mostly two exons) than the coding genes, which may be responsible for the differences in their evolution and function. This result is similar to the angiosperm poplar and gymnosperm Norway spruce [30, 34]. LncRNAs are typically greater than 200 nucleotides in length, whereas only 3% of lncRNA were > 1 kb in length in this ginkgo study. A similar condition was observed in the study of lncRNAs in maize [35]. This indicates that a small number of long lncRNAs exist in plants.

RNA plays not only an auxiliary role as an intermediate carrier of genetic information but also a role in a variety of regulatory functions. LncRNA is essentially RNA, a long chain composed of nucleotides, which can affect the biological activities of eukaryotes through various mechanisms of action [5, 7]. Since the functions of lncRNAs are highly complex and diverse, unlike the mRNA sequences that can provide potential functional information, the sequence motifs of lncRNAs generally do not provide information to predict lncRNA functions [9, 32]. However, lncRNAs may regulate gene expression either in a *cis*- or *trans*-acting manner [36]. The regulatory roles of lncRNAs in gene expression were achieved through acting on the adjacent target genes, and this was known as the cis-acting process of lncRNAs [37–39]. Transposable elements can regulate adjacent gene expression as a *cis*-element [40]. To further analyze the lncRNA function, we have obtained 48 DELs with 72 target genes within a 100 kb range; therefore, these 72 target genes may be regulated by DELs. Because the *trans*-acting lncRNAs regulate gene expression at independent loci [41], 31 DELs regulated 31 different target genes in this study, which are involved in pigment formation process and may be an important reason for leaf-color mutation. In addition, several studies have shown that lncRNAs coordinate miRNAs, forming multiple feedforward pathways to regulate a range of target genes [42, 43]. For example, some lncRNAs can act as precursors for miRNAs [44]. Some studies have proposed that lncRNAs may also function as miRNA primary transcripts, targets, or target mimics, providing a new mechanism for the regulation of miRNA activity [45–47]. Thus, the identification and analysis of the correlation between lncRNAs and miRNA precursors will help elucidate regulatory processes [43]. These results will also help explore the functions of the corresponding lncRNAs [48]. We detected six novel lncRNAs as precursors to known miRNAs that were identified in this study, which lays a foundation for the subsequent study of leaf-color mutations.

LncRNA can participate in the regulation of gene expression through various mechanisms [49–51]. Several lncRNAs can regulate mRNAs by binding or interacting with their targets [28, 52]. This could be the result of the direct regulation of lncRNAs with the promoter region or other *cis*-regulated elements of its coexpressed protein-coding genes [53]. To investigate whether ginkgo lncRNAs have the potential to interact with sequences of their targets, we performed GO and KEGG analysis on *cis*- and *trans*-targets of lncRNAs. The results showed that the chloroplast thylakoid membrane subcategory was most enriched in the CC category and that photosynthesis pathways (ko00195) were most enriched in the KEGG pathway analysis. The chloroplast thylakoid membrane subcategory was highly enriched, which may result in a green-deficient leaf color or lead to an abnormal leaf color [54]. Chlorophyll is an important pigment related to photosynthesis, and leaf-color variations are closely related to pigment synthesis [26, 48]. This suggests that the complex mechanism of leaf-color variation requires a coordinated regulatory network of posttranscriptional gene expression [55]. Although pigment synthesis may be a multifactorial phenotypic trait, only a few pathways regulating pigment synthesis have been validated [28, 56], and our understanding of the role of lncRNA in pigment synthesis is very limited [57]. Hence, we established a substantial number of coexpression modules to reveal the regulatory relationships and functions of leaf-color mutation or pigment synthesis. These coexpression analyses implied the functional correlation of lncRNA and protein coding, especially for TFs (MYB-related and bHLH). LncRNAs interact with a myriad of genes encoding TFs [43]. A previous study indicated that the trans-acting lncRNA HID1 associates with the chromatin of the TF gene PIF3 and can repress its transcription in Arabidopsis [58]. Several studies have shown that lncRNAs can regulate the activity of TFs [59–61]. Studies have indicated that the regulatory factors involved in pigment synthesis include MYB, bHLH and WD40, among which MYB plays the most important role in regulating anthocyanin synthesis, which has been proven in apple and grape species [62, 63]. These results provides a useful source for further research on pigment formation in plants.

## Conclusions

In this study, we obtained 2044 lncRNAs including 238 DELs involved in the ginkgo leaf-color mutation through high-throughput sequencing. The results showed that 48 *cis*-acting DELs might regulate 72 target genes, and 31 *trans*-acting DELs might regulate 31 different target genes. The chloroplast thylakoid membrane subcategory and photosynthesis pathways (ko00195) were most enriched in GO and KEGG analyses. In addition, 32 DELs and 49 DEGs constituted coexpression networks, and eight DELs and 68 TFs had interaction networks. This study will provide a basis for subsequent studies on the molecular biology of the leaf color of ginkgo and will also provide a reference for the study of other plants in related fields.

## Methods

### Plant materials and RNA sequencing

Since ginkgo is listed as “Endangered” on the red list, we first obtained permission to collect ginkgo leaves and branches. Plant materials were collected from about 150-year-old ginkgo tree in Jiujiang city, Jiangxi Province, China (29°49′ N, 116°40’E). The ginkgo leaves exhibited GL and YL phenotypes on a main branch of the tree. And YL phenotype was identified as a xantha mutant (*Ginkgo biloba* “Wannianjin”) by Professor Fuliang Cao. Phenotypes of GL and YL mutant also exhibited in the Additional file 2: Figure S1 of our previous research [27]. In addition, several GL and YL scions were grafted onto rootstocks in the ginkgo germplasm nursery at Nanjing Forestry University Base. These samples were fully expanded mature leaves (free from pests and diseases). Three leaves were sampled per replication with three replicates for each group in the same period. The total RNA from these leaves (GL and YL) was extracted and purified as previously described [23] with a slight modification in the Illumina sequencing platform (HiSeq™ X) used for reference transcriptome sequencing (Ginkgo genome: http://gigadb.org/dataset/100209, [64]).

### Identification of lncRNAs

**Fig. 8:**
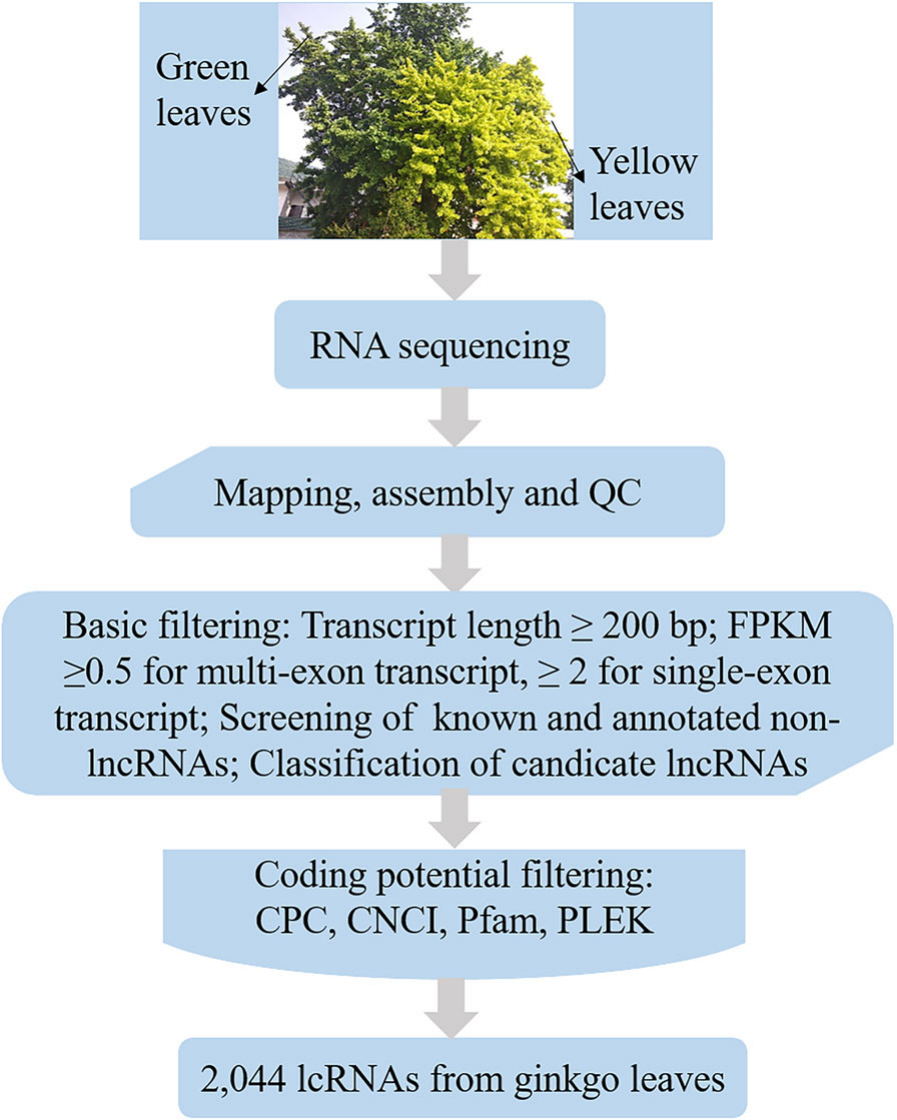
Identification of ginkgo long noncoding RNAs (lncRNAs)

First, each of the reconstructed transcripts was pooled to generate a collection of transcripts representative of transcription using StringTie software [65]. Then, based on the characteristics of lncRNA, we used a strict four-step screening method to obtain candidate lncRNAs. Step 1: Cuffcompare software was used to compare the merged transcripts with reference transcripts one-by-one to clarify the location of the remaining transcripts. Transcripts with the words “I”, “U”, “X”, and “O” were retained by screening the candidate lncRNA transcripts. Step 2: Transcripts greater than 200 bp in length and harboring 2 or more exons were screened and retained. Step 3: The transcripts screened in the second step were predicted and analyzed for their coding capacity, and these transcripts with coding potential were removed. The software used for the characterization of the obtained lncRNA sequences included CPC [66] and CNCI [67] as well as the Pfam [68] and PLEK databases [69]. Step 4: For species with known lncRNAs, the lncRNA sequences obtained in the third step were aligned with the known lncRNA using BLASTN to remove the duplicate sequences. After the known lncRNA sequences were combined, quantitative analysis was performed. Otherwise, the lncRNA sequences obtained in the third step were directly used for quantitative analysis. Furthermore, the transcripts obtained for each sample were merged and compared to known transcripts using Cuffcompare. Finally, the abovementioned candidate lncRNAs were further screened by using the most widely used coding potential analysis methods, including CPC analysis, CNCI analysis, Pfam protein domain analysis, and PLEK analysis. The final lncRNA sequence statistics were obtained (Fig. 8). Moreover, lncRNAs were aligned to the Rfam database using INFERNAL, and the family of lncRNAs was annotated. BLAST was used to map lncRNAs to miRBase for potential miRNA precursors, and lncRNAs with miRNA precursor coverage greater than 90% were selected.

### Expression level annotation and DEL screening

We used a reference transcript as a library, and the abundance of the expression of each transcript in each sample was determined by the method of sequence similarity alignment. Bowtie 2 [70] and expression analysis [71] were used. The transcript expression quantity was calculated using the FPKM method [72]. The number of counts of each sample lncRNA was normalized by DESeq software [73], and the difference multiple was calculated. The difference in the number of reads was tested by negative binomial distribution test. Finally, the DELs were screened according to the different multiple and differential significance test results.

### DEL and DEG coexpression analysis

Transcriptome sequencing assembly and functional annotations were performed according to the methods of Wu et al. (2018) [23]. To identify the DEGs, the two different groups were statistically compared via DESeq software [73]. Specifically, the differential expression were tested via a negative binomial distribution and a shrinkage estimator for the variance of the distribution. The false discovery rate was used as a threshold for the *p*-value for multiple tests to judge the significance of gene expression differences. A Pearson correlation test was used to determine the correlation between the expression data for the DELs and DEGs. The relationship pairs with a correlation coefficient greater than 0.8 and a p-value less than or equal to 0.05 were considered to have a coexpression relationship. Top50 was used to construct the coexpression network.

### Target gene prediction

Because lncRNAs mainly have a *cis*- or *trans*-acting function on target genes, lncRNAs were divided into two cases to predict target genes. On the one hand, all protein-coding genes near the lncRNAs in the upstream and downstream 100 kb and significantly coexpressed with the lncRNA were screened as target genes. On the other hand, the target genes of *trans*-acting lncRNAs were identified by the correlation of expression levels rather than the positional relationships. These lncRNA target genes were functionally annotated using the GO (http://geneontology.org/) and KEGG (http://www.genome.jp/kegg/) databases. Based on the results of the differentially expressed coexpression analysis, lncRNA and mRNA with different chromosomes were selected as candidate targets to extract candidate sequences. The RNA interaction software RIsearch-v2.0 was used to predict the binding of candidate lncRNAs and mRNAs at the nucleic acid level. According to the screening condition, the number of bases between two nucleic acid molecules directly interacting with each other was no less than 10, and the free energy of base binding was no more than 50; the screened lncRNA and mRNA may have direct regulation.

### Correlation analysis of lncRNAs and TFs

For each DEL, the coexpressed coding genes were calculated, and the significance of differential mRNA enrichment in each TF entry was calculated using the hypergeometric distribution test method. The result of the calculation returned a *p*-value that was significant for the enrichment. Then, the intersection of the lncRNA coexpression coding gene set and TF set was calculated. The hypergeometric distribution was used to calculate the enrichment degree of the intersection, and the TFs significantly related to lncRNAs were obtained, thereby identifying the TFs that may play a regulatory role in combination with lncRNAs.

### Real-time quantitative PCR validation

We randomly selected 10 DELs from the results of the transcriptional analysis and confirmed them by qRT-PCR. All qRT-PCR experiments were performed on an ABI ViiA 7 Real-time PCR platform (Applied Biosystems, Carlsbad, CA, USA). All reactions were performed in triplicate. The PCR program was performed according to Xu et al. [74], and the glyceraldehyde-3-phosphate dehydrogenase gene (forward primer [5′-3′]: GGTGCCAAAAAGGTGGTCAT; reverse primer [5′-3′]: CAACAACGAACATGGGAGCAT) was used as a reference gene [23]. All primers for lncRNAs were designed with Oligo v6.0 software and are listed in Additional file 1: Table S1. We normalized the relative expression of the genes with the 2^−ΔΔCt^ method [75].

## Supplementary information

**Additional file 1: Table S1.** Primer pairs for quantitative real-time PCR.

**Additional file 2: Figure S1.** The expression patterns of ten lncRNAs in the leaves of *Ginkgo*. LncRNAs expression were analysed by quantitative real-time PCR (qRT-PCR) (a) and by their values of fragments per kilobase per million reads (FPKM) (b). The error bars represent the standard errors of the means of three independent replicates.

**Additional file 3: Data S1.** Full-length sequences of lncRNAs.

**Additional file 4: Data S2.** Gene expression.

**Additional file 5: Data S3.** Differentially expressed lncRNAs.

**Additional file 6: Data S4.** Differentially expressed mRNAs.

## Data Availability

All raw data from Illumina sequencing including lncRNAs and RNA-seq data have been submitted to the Short Reads Archive database under the accession number SRP182122.

## Abbreviations

BP: Biological process
CC: Cellular component
CNCI: Coding-Non-Coding Index
CPC: Coding Potential Calculator
DEGs: Differentially expressed mRNAs
DELs: Differentially expressed lncRNAs
FPKM: Fragments per kilobase per million reads
Ginkgo: *Ginkgo biloba* L
GL: Normal green-colored leaves
GO: Gene Ontology
I: Intronic lncRNA
KEGG: Kyoto Encyclopedia of Genes and Genomes
lincRNA, U: Intergenic lncRNA
lncRNA: long noncoding RNA
MF: Molecular function
ncRNAs: noncoding RNAs
O: Sense-overlapping lncRNA
Pfam: Protein Families
PLEK: Predictor of long noncoding RNAs and messenger RNAs based on an improved k-mer scheme
qRT-PCR: quantitative real-time PCR
RNA-seq: RNA sequencing
SRA: Short Reads Archive
TFs: Transcription factors
X: Antisense lncRNA
YL: Yellow-colored leaves
