# Supplementary material for: Transcriptional profiling of long noncoding RNAs associated with leaf-color mutation in Ginkgo biloba L
Source: BMC Plant Biol. 2019 Nov 29;19:527. doi: 10.1186/s12870-019-2141-z (PMC6884798; doi:10.1186/s12870-019-2141-z)
Supplement: Supplementary file 1 — Additional file 1: Table S1. Primer pairs for quantitative real-time PCR. [file 12870_2019_2141_MOESM1_ESM.docx]

**Supplementary information**

**Table S1.** Primer pairs for quantitative real-time PCR.

| Name | Forward (5'-3') | Reverse (5'-3') |
| --- | --- | --- |
| lnc1 | ACTGGAAGGTGCGGCTGG | GCTCGTGAACTTGGTCTTAC |
| lnc2 | GGGGTGGGTGGATGCGTG | CCCTCTCCCTATCGGTCG |
| lnc3 | AGTCCTCCCAGAACCAAAAG | CCGAAATGAATGAATAAAGGTG |
| lnc4 | CAGAGTGATAGAGCGTTCGT | CTTCCAACATTTCCACCACC |
| lnc5 | GTTATTACACTGAGAAGGGGA | TTGTGGAGTGAGTTGTGGAGT |
| lnc6 | CAGTGGTCATCCCTATTTTTC | TCCTGCCATAAGAAGTCCAAG |
| lnc7 | GACACAGCAGTTCTCTTCACA | ACACATACCTCTGGACCAAAG |
| lnc8 | GGCAGCAAGAAGGGCAACA | CAGTCGTTCTCCCCACCCA |
| lnc9 | TGTAGTTCATCGTTTCAGGAC | TCATCTCAACAGCTACGTGG |
| lnc10 | CAGTTTCAATCGGGGGGAG | ATTCTTTGTGTCGGATGGCTG |
