# Supplementary figures and images for: Transcriptional profiling of long noncoding RNAs associated with leaf-color mutation in Ginkgo biloba L
Source: BMC Plant Biol. 2019 Nov 29;19:527. doi: 10.1186/s12870-019-2141-z (PMC6884798; doi:10.1186/s12870-019-2141-z)

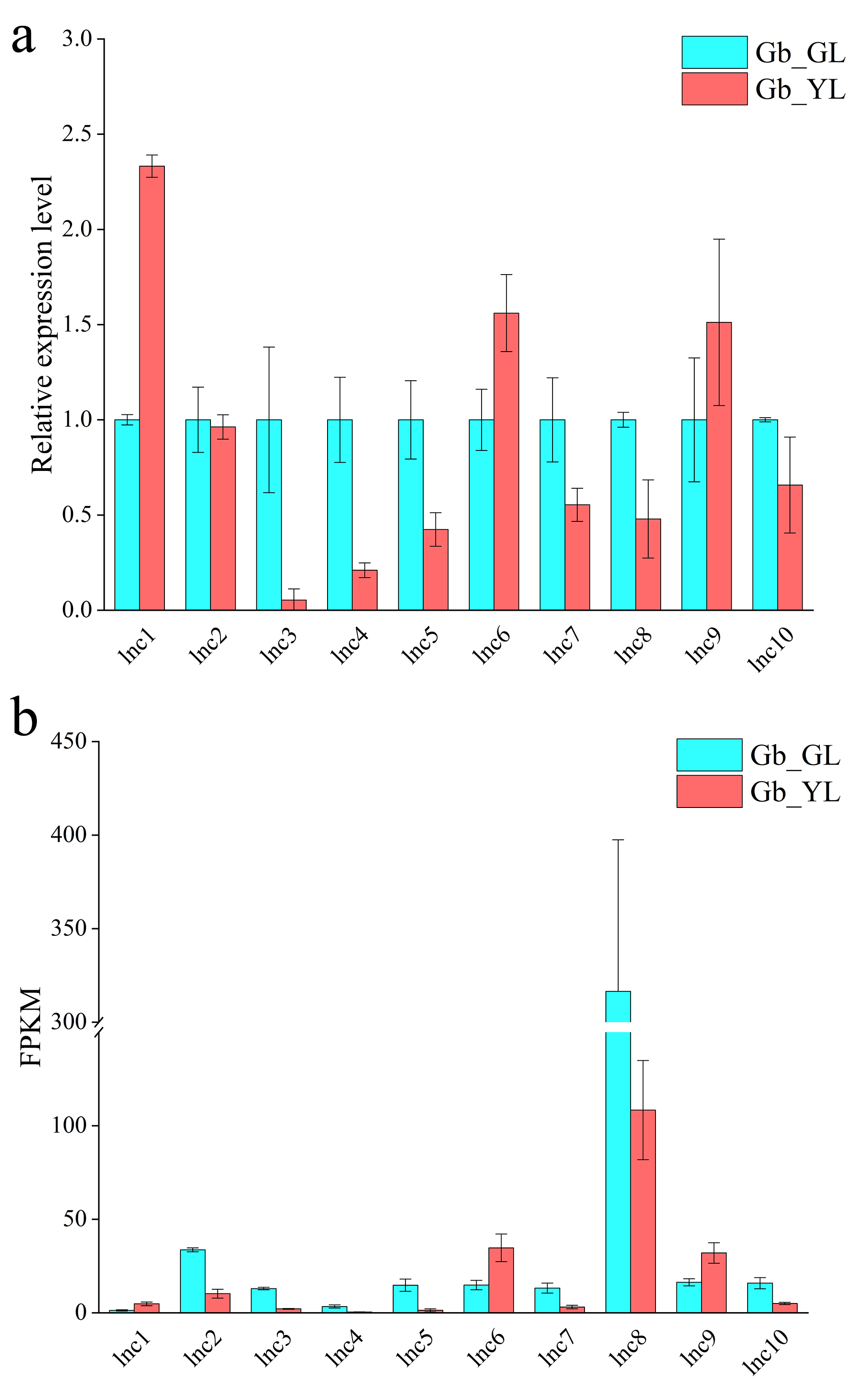

Supplement: Supplementary file 2 — Additional file 2: Figure S1. The expression patterns of ten lncRNAs in the leaves of Ginkgo. LncRNAs expression were analysed by quantitative real-time PCR (qRT-PCR) (a) and by their values of fragments per kilobase per million reads (FPKM) (b). The error bars represent the standard errors of the means of three independent replicates. [file 12870_2019_2141_MOESM2_ESM.tif]
